# Genome-wide CRISPR/Cas9 screen identifies regulators of BCMA expression on multiple myeloma cells

**DOI:** 10.1038/s41408-024-00986-z

**Published:** 2024-01-25

**Authors:** Ram Ajore, Jenny Mattsson, Maroulio Pertesi, Ludvig Ekdahl, Zain Ali, Markus Hansson, Björn Nilsson

**Affiliations:** 1 Division of Hematology and Transfusion Medicine, Department of Laboratory Medicine, Lund University, 221 84 Lund, Sweden; 2 Lund Stem Cell Center, Lund University, 221 84 Lund, Sweden; 3 BioInvent International AB, Ideongatan 1, 223 70 Lund, Sweden; 4 Department of Internal Medicine and Clinical Nutrition, Sahlgrenska Academy, Göteborg University, 41346 Göteborg, Sweden; 5 Broad Institute, Cambridge, MA 02142 USA

**Keywords:** Translational research, Cancer immunotherapy

Multiple myeloma (MM) is a blood malignancy defined by an uncontrolled clonal growth of plasma cells. Currently, new immunotherapies are being introduced that utilize BCMA to redirect the patient’s T-cells to kill MM cells, using either chimeric antigen receptor (CAR) T-cells [1] or bispecific BCMAxCD3 T-cell-engaging antibodies [2, 3]. These agents show unprecedented effects in early clinical trials, including an almost complete initial response rate in patients with refractory MM and durable remissions in ~50% of these patients [1–5]. However, the efficacy depends on the BCMA expression level. Downregulation of BCMA can limit long-term effectiveness and lead to relapse [6–8]. Conversely, cytokine release syndrome has been reported in patients with extremely high levels of BCMA expression on MM cells [9]. Hence, it is essential to understand the regulation of BCMA expression.

BCMA, encoded by *TNFRSF17*, is a receptor surface glycoprotein receptor expressed on plasma cells [10, 11]. Previously, only a few regulators of BCMA expression have been reported. Firstly, the γ-secretase protease cleaves BCMA and about 140 other transmembrane proteins [12], including amyloid precursor protein. γ-secretase inhibitors, originally developed to prevent amyloid formation in Alzheimer’s disease [13], have been suggested as a means to boost BCMA-targeted immunotherapy for MM [14–16]. Secondly, the POU2AF1, PRDM1, IRF4, and RUNX3 transcription factors and the IL4 and IL6 cytokines have been suggested to upregulate BCMA [17–22].

To search for BCMA regulators, we conducted a genome-wide CRISPR/Cas9 screen in the OPM2 and MOLP8 MM cell lines using the Brunello library [23], containing 76,441 small guide RNA (sgRNA) sequences targeting 19,114 genes. We isolated BCMA-high-expressing (BCMA^hi^) and BCMA-low-expressing (BCMA^lo^) cells by fluorescence-activated cell sorting and assessed sgRNA representation using massively parallel sequencing. The details of the experiments are described in Supplementary Methods.

Twenty-six genes showed significant differences in sgRNA representation (Supplementary Table 1). These genes showed a strong correlation between OPM2 and MOLP8 effect sizes (Pearson *r* = 0.70, *P* = 6.0 × 10^−5^). We observed enrichment of *TNFRSF17* in BCMA^lo^ cells (Fig. 1; Supplementary Table 1), confirming specificity. Analysis of bulk- and single-cell mRNA-sequencing data showed enrichment of expression of the 26 genes in plasma cells (Supplementary Figs. 1–3). To see if the identified genes regulate other MM immunotherapy targets, we carried out similar CRISPR/Cas9 screens for CD38 and CD319, observing no convincing effects for any of the 26 genes (Supplementary Table 2).

**Fig. 1:**
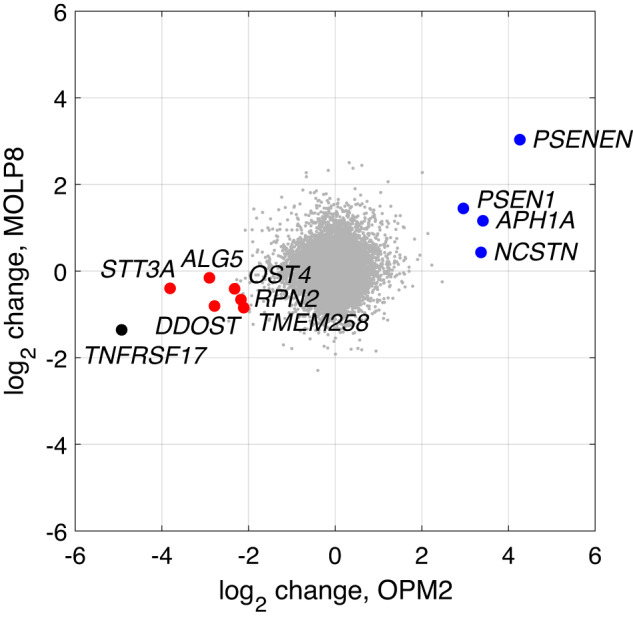
Screening results. We performed genome-wide CRISPR/Cas9 screens in the OPM2 and MOLP8 MM cell lines. We sorted BCMA-high-expressing (BCMA^hi^) and BCMA-low-expressing (BCMA^lo^) cells, determined the sgRNA abundance by massively parallel sequencing, and calculated log_2_ ratios reflecting the sgRNA frequency in BCMA^hi^ relative to BCMA^lo^ cells. The *x* and *y* axes represent OPM2 and MOLP8 cells, respectively. *Blue*: Genes encoding γ-secretase subunits. *Red:* Genes encoding oligosaccharyltransferase subunits or other enzymes involved in N-glycosylation. *Black: TNFSRF17*, which encodes BCMA itself. The summary statistics are given in Supplementary Table 1.

The identified set of genes showed more functional interactions than expected (STRING database [24]; 26 interactions *vs*. seven expected, *P* = 3.4 × 10^−8^), with γ-secretase and oligosaccharyl transferase genes forming distinct subnetworks (Supplementary Fig. 4; Supplementary Table 3). The most enriched gene in BCMA^hi^ cells was *PSENEN*, encoding presenilin enhancer 2, an essential γ-secretase subunit [25, 26]. γ-secretase also contains nicastrin (NCSTN), presenilin 1 or 2 (PSEN1 or PSEN2), and aph1 homolog A or B (APH1A or APH1B). NCSTN is a substrate-recruiting component [27], PSEN1 and PSEN2 alternative active subunits [28], and APH1A and APH1B alternative stabilizing subunits [29–32]. Combinatorically, the incorporation of either APH1A or APH1B and either PSEN1 or PSEN2 produces four types of γ-secretase [31–38]. We found enrichment in BCMA^hi^ cells of *NCSTN*, *PSEN1*, and *APH1A* but not for *PSEN2* and *APH1B* (Fig. 1, Supplementary Table 1, 2). Directed CRISPR/Cas9 knockdown of the four γ-secretase genes identified in the screen increased BCMA expression up to 11.2-fold, whereas knockdown of *APH1B* and *PSEN2* produced weaker effects (Fig. 2 and Supplementary Fig. 5).

**Fig. 2:**
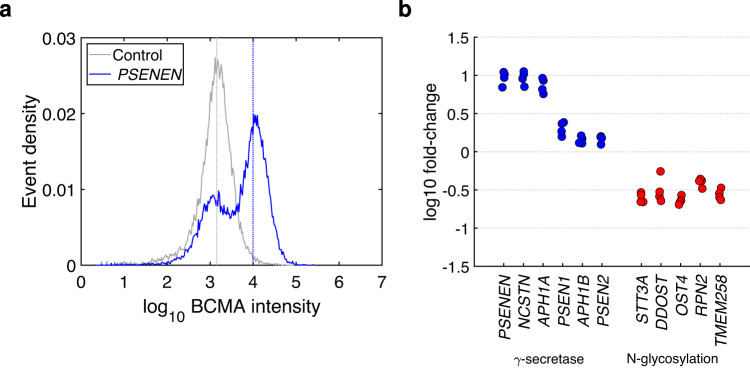
Summary of validation data. To validate our screening results and directly estimate effects on BCMA expression, we performed CRISPR/Cas9 knockdown of 14 genes in OPM2 cells (detailed data in Supplementary Fig. 5). **a** Representative example showing the effects of CRISPR/Cas9 knockdown, in this case of the *PSENEN* gene. CRISPR/Cas9-treated cells show a bimodal distribution (blue), reflecting CRISPR-edited and unedited cells. Untreated cells show a unimodal distribution (grey). Using Gaussian Mixture Modeling, we estimated the mean BCMA intensity of the right-shifted cell population (blue line). We calculated the log10 fold-change relative to the mean intensity of untreated cells (grey line). **b** Summary of changes in BCMA expression for all genes tested x four biological replicates each. *Blue:* Genes encoding γ-secretase subunits. *Red:* Genes encoding oligosaccharyltransferase subunits or other enzymes involved in N-glycosylation.

In BCMA^lo^ cells, we detected enrichment of 6 genes involved in protein N-glycosylation (*STT3A, DDOST, ALG5*, *TMEM258*, *RPN2*, and *OST4*; Fig. 1, Supplementary Table 1). BCMA was recently identified as a glycoprotein with a complex type N-glycan at a single N-glycosylation site, asparagine 42, and altered glycosylation affects BCMA ligand binding [39]. Strikingly, *DDOST*, *STT3A*, *RPN2*, *TMEM258*, and *OST4* all encode subunits of the oligosaccharyltransferase (OST) complex that catalyzes the initial transfer of high-mannose oligosaccharides (Glc(3)Man(9)GlcNAc(2)) to asparagine residues within the Asn-X-Ser/Thr motif, the first step in N-glycosylation [40]. *ALG5* encodes an enzyme required for the addition of glucose residues to the oligomannose core [41]. For further validation, we knocked down five of the N-glycosylation genes by directed CRISPR/Cas9, observing 3.6-fold downregulation of BCMA on average (Fig. 2 and Supplementary Fig. 5). These data indicate that N-glycosylation is required for BCMA presentation on the MM cell surface.

Somatic loss-of-function mutations in genes required for BCMA expression could confer resistance to BCMA-targeted immunotherapies. To understand if loss-of-function mutations in the identified N-glycosylation genes are tolerated by MM cells, we analyzed CRISPR/Cas9 knockdown effects in the DepMap compendium, observing no or only mild suppression of cell growth (median Chronos gene score >−1) for 5 of the 6 N-glycosylation genes (Supplementary Fig. 6). Consistent with this, none of the N-glycosylation genes showed evidence of intolerance to loss-of-function variants in the Genome Aggregation Database (Supplementary Table 4). Additionally, germline loss-of-function mutations in *DDOST* underlie Congenital Disorder of Glycosylation type Ir, an autosomal recessive disorder characterized by developmental defects, intellectual disability, and humoral immunodeficiency [42, 43]. Loss-of-function mutations in *ALG5* have been reported in atypical polycystic kidney disease. These observations suggest that loss-of-function mutations in the N-glycosylation genes are unlikely to lead to clonal elimination.

In addition to γ-secretase genes and N-glycosylation genes, we detected 16 genes significantly affecting BCMA expression. For example, in BCMA^hi^ cells, we saw strong enrichment of *HEXIM1* (HEXIM P-TEFb Complex Subunit 1) and *UBE2M* (ubiquitin-conjugating enzyme E2M). *HEXIM1* functions as an RNA polymerase II inhibitor [44] and regulator of NF-κ-B and corticosteroid-driven transcription [45, 46], which play key roles in MM. *UBE2M* encodes an E2 ubiquitin ligase that attaches ubiquitin to proteins to trigger their degradation. Directed knockdown of *HEXIM1* and *UBE2M* upregulated BCMA 2.3-fold and 3.8-fold, respectively (Supplementary Fig. 5). Interestingly, no ubiquitination mechanism has been described before for BCMA. Additional genes of interest include those implicated in transcriptional regulation (*TP53TG3B*, *POLR1A*, *CNIH1*, *ZNF792*, *TCEB2*), mitochondrial metabolism (*TAZ*, *CO15*), and ribosome biogenesis (*SDAD1*, *LTV1*).

In summary, we report a genome-wide screen for regulators of BCMA expression. Using conservative criteria, we identify 26 genes. Of these, only the four γ-secretase genes belong to a biological process previously implicated in BCMA regulation [14, 15]. These results have potential for clinical translation: Firstly, we confirm γ-secretase as a potent negative regulator of BCMA expression. In a recent phase-1 study, patients with relapsed MM were pre-treated with γ-secretase inhibitor before receiving BCMA CAR T-cells [16], producing an average 12.2-fold upregulation of BCMA, which is on par with our findings. Our data and this trial warrant intensified studies to determine the value of adding γ-secretase inhibitors to BCMA-directed immunotherapy. Secondly, we identify impaired N-glycosylation as a tentative resistance mechanism to BCMA-targeted immunotherapies. Accordingly, these genes should be investigated further in samples of MM patients resistant versus sensitive to BCMA-targeting immunotherapies; such data sets will likely become available once BCMA-targeting agents are used on a larger scale. Finally, we identify several new genes that could potentially be utilized to boost BCMA expression, including several additional regulators with strong effects (*e.g*., *HEXIM1* and *UBE2M*). While detailed investigations of each of these genes are beyond the scope of this study, further studies should be performed to verify the mechanistic impact on the anti-MM activity of T-cell-engaging immunotherapies in both cell lines and primary MM cells. Our work provides new insight into the regulation of BCMA expression, with potential implications for the treatment of MM.

### Supplementary information

Supplementary Methods

Supplementary Figures and Tables

## Data Availability

The raw sequencing data from our CRISPR/Cas9 screens in MOLP8 and OPM2 cells have been deposited in the Sequence Read Archive (SRA; accession number PRJNA1043457).

## References

[1] Berdeja JG, Madduri D, Usmani SZ, Jakubowiak A, Agha M, Cohen AD (2021). Ciltacabtagene autoleucel, a B-cell maturation antigen-directed chimeric antigen receptor T-cell therapy in patients with relapsed or refractory multiple myeloma (CARTITUDE-1): a phase 1b/2 open-label study. Lancet.

[2] Usmani SZ, Garfall AL, van de Donk N, Nahi H, San-Miguel JF, Oriol A (2021). Teclistamab, a B-cell maturation antigen x CD3 bispecific antibody, in patients with relapsed or refractory multiple myeloma (MajesTEC-1): a multicentre, open-label, single-arm, phase 1 study. Lancet.

[3] Moreau P, Garfall AL, van de Donk N, Nahi H, San-Miguel JF, Oriol A (2022). Teclistamab in relapsed or refractory multiple myeloma. N. Engl J Med.

[4] Donk NWCJVD, Moreau P, Garfall AL, Bhutani M, Oriol A, Nooka AK (2023). Long-term follow-up from MajesTEC-1 of teclistamab, a B-cell maturation antigen (BCMA) x CD3 bispecific antibody, in patients with relapsed/refractory multiple myeloma (RRMM). J Clin Oncol.

[5] Lin Y, Martin TG, Usmani SZ, Berdeja JG, Jakubowiak AJ, Agha ME (2023). CARTITUDE-1 final results: Phase 1b/2 study of ciltacabtagene autoleucel in heavily pretreated patients with relapsed/refractory multiple myeloma. J Clin Oncol.

[6] Brudno JN, Maric I, Hartman SD, Rose JJ, Wang M, Lam N (2018). T cells genetically modified to express an anti-b-cell maturation antigen chimeric antigen receptor cause remissions of poor-prognosis relapsed multiple myeloma. J Clin Oncol.

[7] Pont MJ, Hill T, Cole GO, Abbott JJ, Kelliher J, Salter AI (2019). gamma-Secretase inhibition increases efficacy of BCMA-specific chimeric antigen receptor T cells in multiple myeloma. Blood.

[8] van de Donk N, Themeli M, Usmani SZ (2021). Determinants of response and mechanisms of resistance of CAR T-cell therapy in multiple myeloma. Blood Cancer Discov.

[9] Li D, Que Y, Ding S, Hu G, Wang W, Mao X (2022). Anti-BCMA CAR-T cells therapy for a patient with extremely high membrane BCMA expression: a case report. J Immunother Cancer.

[10] Frigyesi I, Adolfsson J, Ali M, Christophersen MK, Johnsson E, Turesson I (2014). Robust isolation of malignant plasma cells in multiple myeloma. Blood.

[11] Tai YT, Acharya C, An G, Moschetta M, Zhong MY, Feng X (2016). APRIL and BCMA promote human multiple myeloma growth and immunosuppression in the bone marrow microenvironment. Blood.

[12] Guner G, Lichtenthaler SF (2020). The substrate repertoire of gamma-secretase/presenilin. Semin Cell Dev Biol.

[13] Hur JY (2022). gamma-Secretase in Alzheimer’s disease. Exp Mol Med.

[14] Metelo AM, Jozwik A, Luong LA, Dominey-Foy D, Graham C, Attwood C (2022). Allogeneic Anti-BCMA CAR T cells are superior to multiple myeloma-derived CAR T cells in preclinical studies and may be combined with gamma secretase inhibitors. Cancer Res Commun.

[15] Chen H, Yu T, Lin L, Xing L, Cho SF, Wen K (2022). gamma-secretase inhibitors augment efficacy of BCMA-targeting bispecific antibodies against multiple myeloma cells without impairing T-cell activation and differentiation. Blood Cancer J.

[16] Cowan AJ, Pont MJ, Sather BD, Turtle CJ, Till BG, Libby EN (2023). gamma-Secretase inhibitor in combination with BCMA chimeric antigen receptor T-cell immunotherapy for individuals with relapsed or refractory multiple myeloma: a phase 1, first-in-human trial. Lancet Oncol.

[17] Deng S, Yuan T, Cheng X, Jian R, Jiang J (2010). B-lymphocyte-induced maturation protein1 up-regulates the expression of B-cell maturation antigen in mouse plasma cells. Mol Biol Rep.

[18] Zhao C, Inoue J, Imoto I, Otsuki T, Iida S, Ueda R (2008). POU2AF1, an amplification target at 11q23, promotes growth of multiple myeloma cells by directly regulating expression of a B-cell maturation factor, TNFRSF17. Oncogene.

[19] Brady G, Whiteman HJ, Spender LC, Farrell PJ (2009). Downregulation of RUNX1 by RUNX3 requires the RUNX3 VWRPY sequence and is essential for Epstein-Barr virus-driven B-cell proliferation. J Virol.

[20] Shaffer AL, Emre NC, Lamy L, Ngo VN, Wright G, Xiao W (2008). IRF4 addiction in multiple myeloma. Nature.

[21] Klein U, Casola S, Cattoretti G, Shen Q, Lia M, Mo T (2006). Transcription factor IRF4 controls plasma cell differentiation and class-switch recombination. Nat Immunol.

[22] Yang M, Hase H, Legarda-Addison D, Varughese L, Seed B, Ting AT (2005). B cell maturation antigen, the receptor for a proliferation-inducing ligand and B cell-activating factor of the TNF family, induces antigen presentation in B cells. J Immunol.

[23] Doench JG, Fusi N, Sullender M, Hegde M, Vaimberg EW, Donovan KF (2016). Optimized sgRNA design to maximize activity and minimize off-target effects of CRISPR-Cas9. Nat Biotechnol.

[24] Szklarczyk D, Kirsch R, Koutrouli M, Nastou K, Mehryary F, Hachilif R (2023). The STRING database in 2023: protein-protein association networks and functional enrichment analyses for any sequenced genome of interest. Nucleic acids Res.

[25] Haapasalo A, Kovacs DM (2011). The many substrates of presenilin/gamma-secretase. J Alzheimers Dis.

[26] Kopan R, Ilagan MX (2004). Gamma-secretase: proteasome of the membrane?. Nat Rev Mol Cell Biol.

[27] Shah S, Lee SF, Tabuchi K, Hao YH, Yu C, LaPlant Q (2005). Nicastrin functions as a gamma-secretase-substrate receptor. Cell.

[28] Bammens L, Chavez-Gutierrez L, Tolia A, Zwijsen A, De Strooper B (2011). Functional and topological analysis of Pen-2, the fourth subunit of the gamma-secretase complex. J Biol Chem.

[29] Li YM, Lai MT, Xu M, Huang Q, DiMuzio-Mower J, Sardana MK (2000). Presenilin 1 is linked with gamma-secretase activity in the detergent solubilized state. Proc Natl Acad Sci USA.

[30] Steiner H, Haass C (2000). Intramembrane proteolysis by presenilins. Nat Rev Mol Cell Biol.

[31] Kimberly WT, LaVoie MJ, Ostaszewski BL, Ye W, Wolfe MS, Selkoe DJ (2003). Gamma-secretase is a membrane protein complex comprised of presenilin, nicastrin, Aph-1, and Pen-2. Proc Natl Acad Sci USA.

[32] De Strooper B (2003). Aph-1, Pen-2, and Nicastrin with Presenilin generate an active gamma-Secretase complex. Neuron.

[33] Francis R, McGrath G, Zhang J, Ruddy DA, Sym M, Apfeld J (2002). aph-1 and pen-2 are required for Notch pathway signaling, gamma-secretase cleavage of betaAPP, and presenilin protein accumulation. Dev Cell.

[34] Goutte C, Tsunozaki M, Hale VA, Priess JR (2002). APH-1 is a multipass membrane protein essential for the Notch signaling pathway in Caenorhabditis elegans embryos. Proc Natl Acad Sci USA.

[35] Pinnix I, Council JE, Roseberry B, Onstead L, Mallender W, Sucic J (2001). Convertases other than furin cleave beta-secretase to its mature form. FASEB J.

[36] Jurisch-Yaksi N, Sannerud R, Annaert W (2013). A fast growing spectrum of biological functions of gamma-secretase in development and disease. Biochim et Biophys acta.

[37] Acx H, Chavez-Gutierrez L, Serneels L, Lismont S, Benurwar M, Elad N (2014). Signature amyloid beta profiles are produced by different gamma-Secretase Complexes. J Biol Chem.

[38] Sannerud R, Esselens C, Ejsmont P, Mattera R, Rochin L, Tharkeshwar AK (2016). Restricted location of PSEN2/gamma-Secretase determines substrate specificity and generates an intracellular Abeta Pool. Cell.

[39] Huang HW, Chen CH, Lin CH, Wong CH, Lin KI (2013). B-cell maturation antigen is modified by a single N-glycan chain that modulates ligand binding and surface retention. Proc Natl Acad Sci USA.

[40] Ramirez AS, Kowal J, Locher KP (2019). Cryo-electron microscopy structures of human oligosaccharyltransferase complexes OST-A and OST-B. Science.

[41] Huffaker TC, Robbins PW (1983). Yeast mutants deficient in protein glycosylation. Proc Natl Acad Sci USA.

[42] Jones MA, Ng BG, Bhide S, Chin E, Rhodenizer D, He P (2012). DDOST mutations identified by whole-exome sequencing are implicated in congenital disorders of glycosylation. Am J Hum Genet.

[43] Sitek A, Ligezka A, Budhraja R, Morava E, Chiarella SE (2023). Pathogenic DDOST variant is associated with humoral immune deficiency. J Clin Immunol.

[44] Yik JH, Chen R, Nishimura R, Jennings JL, Link AJ, Zhou Q (2003). Inhibition of P-TEFb (CDK9/Cyclin T) kinase and RNA polymerase II transcription by the coordinated actions of HEXIM1 and 7SK snRNA. Mol cell.

[45] Ouchida R, Kusuhara M, Shimizu N, Hisada T, Makino Y, Morimoto C (2003). Suppression of NF-kappaB-dependent gene expression by a hexamethylene bisacetamide-inducible protein HEXIM1 in human vascular smooth muscle cells. Genes Cells.

[46] Shimizu N, Ouchida R, Yoshikawa N, Hisada T, Watanabe H, Okamoto K (2005). HEXIM1 forms a transcriptionally abortive complex with glucocorticoid receptor without involving 7SK RNA and positive transcription elongation factor b. Proc Natl Acad Sci USA.

