# Supplementary material for: Genome-wide CRISPR/Cas9 screen identifies regulators of BCMA expression on multiple myeloma cells
Source: Blood Cancer J. 2024 Jan 25;14(1):21. doi: 10.1038/s41408-024-00986-z (PMC10811322; doi:10.1038/s41408-024-00986-z)
Supplement: Supplementary file 1 — Supplementary Methods [file 41408_2024_986_MOESM1_ESM.docx]

**SUPPLEMENTARY METHODS**

**CRISPR/Cas9 screening**

Approximately 400 million OPM2 and MOLP8 cells (DSMZ no. ACC-50 and ACC-569) were transduced with the Brunello library (Addgene no. 73179)^1^ at 0.25 multiplicity-of-infection and 1,000x sgRNA representation. The library contains 77,441 sgRNAs (4 per gene) and 1000 non-targeting control sgRNAs^1^. Spin infections were performed in a 12-well plate at 2,300 rpm for 30 min at 37^0^C for 3-4 million cells per well and 8 ug/ml polybrene (Sigma no. 107689-10g). After 24 hours, transduced cells were selected with puromycin selection for 72 hours. One hundred million cells were stained with phycoerythrin (PE)-conjugated antibodies to BCMA (Rndsystems no. FAB193P), CD38 (BDBiosciences no. 562444), or CD319 (BD Biosciences no. 331805) for 30 min. Three transductions were performed per antibody. BCMA-high-expressing (BCMA^hi^; 5% brightest) and BCMA-low-expressing (BCMA^lo^; 5% darkest) cells were isolated by fluorescence-activated cell sorting (BD FACS Aria Fusion). 2-3 million cells were collected, DNA was extracted (Qiagen no. 69504), and the sgRNA sequence representation was determined by massively parallel sequencing (Novaseq 6000).

**Directed CRISPR/Cas9 knockdown**

To validate and quantify direct effects on BCMA expression, we performed directed CRISPR/Cas9 in OPM2 cells. These sgRNA sequences were cloned into pSpCas9(BB)-2A-GFP (PX458) vector by Gibson assembly (NEBuilder HIFI kit, no. E5520S): *PSENEN* (CCTGGAGCGAGTGTCCAATG), *APH1A* (GACACCACTGATGATACCGA), *NCSTN* (ATGGTCTACGATATGGAGAA), *PSEN1* (GCCACGCAGTCCATTCAGGG), *ALG5* (ATCACTTCATAAGTGAACGC), *APH1B* (GATGATACGCAACGGCTCGG), *DDOST* (CCAACACCTCGAAAGAGGAT), *HEXIM1* (CATGGATGATCACGACCAGG), *OST4* (GAGCGACACGCCCAGCATGT), *PSEN2* (ACTGAGGACACACCCTCGGT), *RPN2* (GCACAACCACAACTGGCACG), *STT3A* (ACAGACATTCCGAATGTCGA), *TMEM258* (AAAGCACCACGGTCAGATGG), *UBE2M* (GCGCAGCTGCGGATCCAGAA). Vectors were transfected into OPM2 cells (Thermofisher Neon). BCMA expression was quantified in GFP-positive cells by flow cytometry using an anti-hBCMA/TNFRSF17-PE antibody (R&D systems no. FAB193P). Editing was confirmed using Sanger sequencing and Synthego ICE (<https://ice.synthego.com>).

**Statistical analysis**

To identify genes differentially represented in BCMA^hi^ and BCMA^lo^ cells, we calculated the overall average normalized sgRNA counts across all sgRNAs targeting the gene and across all replicates. Based on this, we then calculated log2 ratios representing the representation of sgRNAs targeting the gene in BCMA^hi^ cells relative to BCMA^lo^ cells. To assess statistical significance, we calculated false discovery rates using a null distribution consisting of gene scores for null genes generated by randomly selecting the same number of non-targeting sgRNA sequences as for gene-targeting sgRNAs in the library. To estimate the proportions of edited cells and BCMA expression, we fitted a two-component Gaussian Mixture Model to flow cytometry data using maximum likelihood optimization. For robustness, we assumed equal variances between the two components. Genes showing false discovery rates < 1% and absolute log_2_ fold-change > 2 in OPM2 or MOLP8 cells and effects in the same direction in both cell lines were considered significant.

**REFERENCES**

1. Sanson, K.R. *et al.* Optimized libraries for CRISPR-Cas9 genetic screens with multiple modalities. *Nat Commun* **9**, 5416 (2018).
