# Supplementary material for: Genome-wide CRISPR/Cas9 screen identifies regulators of BCMA expression on multiple myeloma cells
Source: Blood Cancer J. 2024 Jan 25;14(1):21. doi: 10.1038/s41408-024-00986-z (PMC10811322; doi:10.1038/s41408-024-00986-z)

Supplementary Table 1

Top genes in CRISPR/Cas9 screen. Positive log_2 ratios indicate enrichment in $BCMA^{hi}$ cells; negative indicate enrichment in $BCMA^{lo}$ cells. Selected genes show q-value < 1% and absolute log_2 fold-change > 2 in either OPM2 or MOLP8 cells, with effects in the same direction in both cell lines. Using these conservative criteria, we identified 26 genes. Blue: genes encoding γ-secretase subunits. Red: genes involved in protein N-glycosylation.

	**OPM2**		**MOLP8**		**Combined**	
**Gene**	**log2 fc**	**q-value**	**log2 fc**	**q-value**	**log2 ratio**	**Function**
PSENEN	4.26	0.0000	3.03	0.0000	3.65	Gamma-secretase subunit
APH1A	3.41	0.0000	1.16	0.0054	2.29	Gamma-secretase subunit
PSEN1	2.96	0.0000	1.45	0.0025	2.20	Gamma-secretase subunit
HEXIM1	2.01	0.0000	2.27	0.0000	2.14	Transcriptional repressor
NCSTN	3.37	0.0000	0.43	0.4192	1.90	Gamma-secretase subunit
UBE2M	1.22	0.0135	2.06	0.0000	1.64	Ubiquitin conjugating enzyme
TP53TG3B	0.61	0.1277	2.44	0.0000	1.53	TP53-Inducible gene 3 protein
PTPN11	2.14	0.0000	0.87	0.0430	1.50	Protein tyrosine phosphatase
DAP	0.32	0.2276	2.5	0.0000	1.41	Programmed cell death
POLR1A	0.41	0.1544	2.37	0.0000	1.39	RNA polymerase I subunit
CNIH1	2.23	0.0000	0.14	0.8846	1.18	ER and Golgi transport
ZNF792	-2.07	0.0000	-0.16	0.8506	-1.12	Transcriptional regulation
TCEB2	-2.18	0.0000	-0.23	0.7632	-1.20	Transcriptional elongation
TAZ	-2.27	0.0000	-0.2	0.8043	-1.24	Mitochondrial phospholipid metabolism
COX15	-2.27	0.0000	-0.3	0.6450	-1.29	Mitochondrial cytochrome C oxidase
SDAD1	-2.13	0.0000	-0.52	0.2867	-1.32	Ribosome biogenesis
LTV1	-0.4	0.1604	-2.29	0.0000	-1.35	Ribosome biogenesis
OST4	-2.32	0.0000	-0.41	0.4565	-1.37	Oligosaccharyltransferase subunit
DNA2	-2.15	0.0000	-0.59	0.2157	-1.37	DNA helicase
RPN2	-2.18	0.0000	-0.66	0.1529	-1.42	Oligosaccharyltransferase subunit
TMEM258	-2.11	0.0000	-0.84	0.0499	-1.48	Oligosaccharyltransferase subunit
ALG5	-2.9	0.0000	-0.16	0.8605	-1.53	Oligosaccharyltransferase subunit
DDOST	-2.79	0.0000	-0.81	0.0642	-1.80	Oligosaccharyltransferase subunit
ATP5B	-2.46	0.0000	-1.15	0.0056	-1.81	ATP synthetase
STT3A	-3.81	0.0000	-0.4	0.4753	-2.11	Oligosaccharyltransferase subunit
TNFRSF17	-4.93	0.0000	-1.36	0.0029	-3.14	Encodes BCMA

Supplementary Table 2

Effects of the top 26 genes identified in the BCMA screen in additional genome-wide, flow-cytometry-based CRISPR/Cas9 screens for regulators of CD38 and CD319.

	**CD38**				**CD319**			
**target_id**	**OPM2_log2**	**OPM2_q**	**MOLP8_log2**	**MOLP8_q**	**OPM2_log2**	**OPM2_q**	**MOLP8_log2**	**MOLP8_q**
PSENEN	-1.420	0.00226	0.616	0.16559	0.235	0.61098	0.41	0.99994
APH1A	0.184	0.55833	1.406	0.00592	0.839	0.18410	0.72	0.44395
PSEN1	0.147	0.65140	-0.041	0.94774	-0.281	0.52695	-0.08	0.99994
HEXIM1	0.344	0.23227	-0.059	0.92277	-0.093	0.86640	0.93	0.19797
NCSTN	-0.707	0.09509	0.691	0.14143	1.000	0.18410	-0.10	0.99994
UBE2M	-0.141	0.66816	-0.351	0.39313	1.487	0.02640	-0.12	0.99994
TP53TG3B	0.507	0.10430	-0.304	0.46899	1.149	0.11548	0.27	0.99994
PTPN11	-0.994	0.04392	0.146	0.76951	-0.368	0.37944	0.92	0.20470
DAP	-0.244	0.41511	1.075	0.02610	1.414	0.02903	0.84	0.26012
POLR1A	-0.139	0.67361	0.573	0.18116	0.784	0.18410	0.08	0.99994
CNIH1	-0.248	0.40537	-0.024	0.97102	-0.126	0.81201	0.37	0.99994
ZNF792	-0.760	0.09509	0.449	0.27114	0.532	0.20817	0.28	0.99994
TCEB2	-0.672	0.09509	0.762	0.12082	0.228	0.62452	0.34	0.99994
TAZ	0.564	0.09607	-0.212	0.63960	-0.742	0.18410	-0.30	0.99994
COX15	0.269	0.36398	0.145	0.77191	-0.317	0.46084	0.17	0.99994
SDAD1	0.390	0.17907	0.073	0.89891	1.016	0.18410	0.35	0.99994
LTV1	0.151	0.64172	0.764	0.12082	0.095	0.86463	0.15	0.99994
OST4	-0.093	0.79234	0.211	0.64341	0.243	0.59646	0.26	0.99994
DNA2	0.381	0.18814	0.529	0.20427	0.787	0.18410	0.86	0.24840
RPN2	-0.774	0.09509	1.191	0.01588	1.307	0.04304	-0.81	0.29620
TMEM258	-1.109	0.01756	1.295	0.01032	1.012	0.18410	-0.14	0.99994
ALG5	0.540	0.09725	0.253	0.56151	-0.379	0.36247	-0.09	0.99994
DDOST	-0.244	0.41463	-0.292	0.49250	-0.861	0.18410	-0.13	0.99994
ATP5B	0.133	0.68925	-0.698	0.13970	0.991	0.18410	1.20	0.11213
STT3A	0.267	0.36905	-0.091	0.86860	-0.377	0.36451	-0.53	0.90809
TNFRSF17	0.079	0.82776	-0.232	0.60057	-0.320	0.45493	0.36	0.99994

Supplementary Table 3

Enriched gene sets identified using the STRING database.

Category	Description	q-value	Genes
GO Process	Protein N-linked glycosylation	3.2E-06	*RPN2,ALG5,STT3A,DDOST,TMEM258,OST4*
GO Process	Protein N-linked glycosylation via asparagine	3.2E-06	*RPN2,ALG5,STT3A,DDOST,OST4*
GO Process	Notch receptor processing	5.6E-06	*NCSTN,PSEN1,APH1A,PSENEN*
GO Process	Amyloid-beta formation	5.6E-06	*NCSTN,PSEN1,APH1A,PSENEN*
GO Process	Membrane protein intracellular domain proteolysis	9.5E-06	*NCSTN,PSEN1,APH1A,PSENEN*
GO Process	Protein glycosylation	2.2E-05	*RPN2,ALG5,PSEN1,STT3A,DDOST,TMEM258,OST4*
GO Process	Membrane protein ectodomain proteolysis	4.5E-05	*NCSTN,PSEN1,APH1A,PSENEN*
GO Process	Organonitrogen compound biosynthetic process	2.5E-04	*COX15,RPN2,ALG5,ATP5F1B,ELOB,NCSTN,PSEN1,STT3A*[illegible]
GO Process	Carbohydrate derivative biosynthetic process	4.2E-04	*RPN2,ALG5,ATP5F1B,PSEN1,STT3A,DDOST,TMEM258,O*[illegible]
GO Process	Organic substance biosynthetic process	1.6E-03	*COX15,RPN2,ALG5,ATP5F1B,ELOB,POLR1A,NCSTN,PSE*[illegible]
GO Process	Cellular macromolecule biosynthetic process	2.7E-03	*RPN2,ALG5,ELOB,PSEN1,STT3A,DDOST,TMEM258,OST4*[illegible]
GO Process	Macromolecule biosynthetic process	2.8E-03	*RPN2,ALG5,ELOB,POLR1A,NCSTN,PSEN1,STT3A,DDOST*[illegible]
GO Process	Positive regulation of proteolysis	4.4E-03	*ELOB,NCSTN,PSEN1,APH1A,DAP,PSENEN*
GO Process	Cellular biosynthetic process	6.7E-03	*COX15,RPN2,ALG5,ATP5F1B,ELOB,POLR1A,PSEN1,STT3*[illegible]
GO Process	Notch signaling pathway	1.0E-02	*NCSTN,PSEN1,APH1A,PSENEN*
GO Process	Positive regulation of endopeptidase activity	4.1E-02	*NCSTN,APH1A,DAP,PSENEN*
GO Component	Oligosaccharyltransferase complex	5.0E-08	*RPN2,STT3A,DDOST,TMEM258,OST4*
GO Component	Gamma-secretase complex	6.7E-07	*NCSTN,PSEN1,APH1A,PSENEN*
GO Component	Endoplasmic reticulum membrane	4.2E-05	*CNIH1,RPN2,ALG5,NCSTN,PSEN1,APH1A,STT3A,DDOST,*[illegible]
GO Component	Membrane protein complex	3.0E-04	*RPN2,ATP5F1B,NCSTN,PSEN1,APH1A,STT3A,DDOST,TM*[illegible]
GO Component	Integral component of presynaptic membrane	6.4E-04	*NCSTN,PSEN1,APH1A,PSENEN*
GO Component	Organelle membrane	5.9E-03	*COX15,CNIH1,RPN2,ALG5,ATP5F1B,NCSTN,PSEN1,APH1*[illegible]
GO Component	Azurophil granule membrane	8.0E-03	*NCSTN,PSEN1,DDOST*
GO Component	Transferase complex	8.1E-03	*RPN2,ELOB,POLR1A,STT3A,DDOST,TMEM258,OST4*
GO Component	Intracellular membrane-bounded organelle	1.1E-02	*COX15,CNIH1,RPN2,ALG5,UBE2M,ATP5F1B,ELOB,POLR1*[illegible]
GO Component	Protein-containing complex	2.0E-02	*COX15,RPN2,ATP5F1B,ELOB,POLR1A,NCSTN,PSEN1,HE*[illegible]
STRING clusters	Oligosaccharyltransferase complex	2.9E-08	*RPN2,STT3A,DDOST,TMEM258,OST4*
STRING clusters	Gamma-secretase complex	9.6E-07	*NCSTN,PSEN1,APH1A,PSENEN*
STRING clusters	Oligosaccharyltransferase complex	1.3E-02	*RPN2,DDOST*
KEGG	N-Glycan biosynthesis	1.9E-04	*RPN2,ALG5,STT3A,DDOST*

KEGG	Notch signaling pathway	1.9E-04	*NCSTN,PSEN1,APH1A,PSENEN*
KEGG	Various types of N-glycan biosynthesis	2.1E-03	*RPN2,STT3A,DDOST*
KEGG	Alzheimer disease	6.4E-03	*ATP5F1B,NCSTN,PSEN1,APH1A,PSENEN*
Reactome	Noncanonical activation of NOTCH3	2.3E-06	*NCSTN,PSEN1,APH1A,PSENEN*
Reactome	Regulated proteolysis of p75NTR	3.1E-06	*NCSTN,PSEN1,APH1A,PSENEN*
Reactome	NOTCH4 Activation and Transmission of Signal	3.1E-06	*NCSTN,PSEN1,APH1A,PSENEN*
Reactome	NRIF signals cell death from the nucleus	5.5E-06	*NCSTN,PSEN1,APH1A,PSENEN*
Reactome	NOTCH2 Activation and Transmission of Signal	1.3E-05	*NCSTN,PSEN1,APH1A,PSENEN*
Reactome	Nuclear signaling by ERBB4	3.3E-05	*NCSTN,PSEN1,APH1A,PSENEN*
Reactome	Activated NOTCH1 Transmits Signal to the Nucleus	3.3E-05	*NCSTN,PSEN1,APH1A,PSENEN*
Reactome	EPH-ephrin mediated repulsion of cells	1.4E-04	*NCSTN,PSEN1,APH1A,PSENEN*
Reactome	Constitutive Signaling by NOTCH1 PEST Mutants	2.0E-04	*NCSTN,PSEN1,APH1A,PSENEN*
Reactome	Constitutive Signaling by NOTCH1 HD+PEST Mutants	2.0E-04	*NCSTN,PSEN1,APH1A,PSENEN*
Reactome	Maturation of spike protein	1.7E-03	*RPN2,STT3A,DDOST*
Reactome	Disease	2.2E-03	*RPN2,ELOB,NCSTN,PSEN1,DNA2,APH1A,STT3A,DDOST,*[illegible]
Reactome	Asparagine N-linked glycosylation	3.4E-03	*CNIH1,RPN2,ALG5,STT3A,DDOST*
Reactome	Axon guidance	4.7E-03	*ELOB,NCSTN,PSEN1,APH1A,PSENEN,PTPN11*
Reactome	Metabolism of proteins	5.0E-03	*CNIH1,RPN2,ALG5,UBE2M,ELOB,NCSTN,APH1A,STT3A,D*[illegible]
Reactome	Amyloid fiber formation	1.0E-02	*NCSTN,APH1A,PSENEN*
Reactome	Diseases of growth factor receptor signaling	1.3E-02	*NCSTN,PSEN1,APH1A,PSENEN,PTPN11*
Reactome	Signaling by Receptor Tyrosine Kinases	3.0E-02	*NCSTN,PSEN1,APH1A,PSENEN,PTPN11*
Reactome	SARS-CoV-2 Infection	3.2E-02	*RPN2,STT3A,DDOST,PTPN11*
Reactome	Signaling by CSF3 (G-CSF)	4.3E-02	*ELOB,PTPN11*
WikiPathways	Inclusion body myositis	5.1E-04	*NCSTN,PSEN1,PSENEN*
WikiPathways	DYRK1A	5.1E-04	*NCSTN,PSEN1,APH1A,PSENEN*
WikiPathways	Notch signaling pathway	5.1E-04	*NCSTN,PSEN1,APH1A,PSENEN*
WikiPathways	Notch signaling	5.8E-03	*NCSTN,PSEN1,APH1A*
WikiPathways	Alzheimers disease and miRNA effects	5.0E-02	*NCSTN,PSEN1,APH1A,PSENEN*
WikiPathways	Alzheimers disease	5.0E-02	*NCSTN,PSEN1,APH1A,PSENEN*
DISEASES	Hidradenitis suppurativa	1.6E-04	*NCSTN,PSEN1,PSENEN*
TISSUES	Anterior pharynx	1.0E-06	*NCSTN,PSEN1,APH1A,PSENEN*
TISSUES	Alimentary canal	1.2E-02	*RPN2,ATP5F1B,POLR1A,NCSTN,PSEN1,DNA2,APH1A,ST*[illegible]
COMPARTMENTS	Oligosaccharyltransferase complex	4.2E-08	*RPN2,STT3A,DDOST,TMEM258,OST4*
COMPARTMENTS	Gamma-secretase complex	4.2E-06	*NCSTN,PSEN1,APH1A,PSENEN*
COMPARTMENTS	Intracellular membrane-bounded organelle	1.5E-03	*COX15,CNIH1,RPN2,UBE2M,ATP5F1B,ELOB,POLR1A,NC*[illegible]

COMPARTMENTS	Membrane protein complex	1.7E-03	*RPN2,ATP5F1B,NCSTN,PSEN1,APH1A,STT3A,DDOST,TM[illegible]*
COMPARTMENTS	Transferase complex	4.5E-03	*RPN2,ELOB,POLR1A,HEXIM1,STT3A,DDOST,TMEM258,O[illegible]*
COMPARTMENTS	Organelle membrane	1.6E-02	*COX15,CNIH1,RPN2,ATP5F1B,NCSTN,PSEN1,STT3A,DD[illegible]*
COMPARTMENTS	Azurophil granule membrane	1.6E-02	*NCSTN,PSEN1,DDOST*
COMPARTMENTS	Nuclear membrane-endoplasmic reticulum membrane	1.6E-02	*RPN2,PSEN1,STT3A,DDOST,TMEM258,OST4*
COMPARTMENTS	Protein-containing complex	2.5E-02	*COX15,RPN2,ATP5F1B,ELOB,POLR1A,NCSTN,PSEN1,HE[illegible]*
COMPARTMENTS	Catalytic complex	2.8E-02	*RPN2,ELOB,POLR1A,HEXIM1,STT3A,DDOST,TMEM258,O[illegible]*
UniProt Keywords	Endoplasmic reticulum	4.1E-04	*CNIH1,RPN2,ALG5,PSEN1,APH1A,STT3A,DDOST,TMEM2[illegible]*
UniProt Keywords	Notch signaling pathway	4.1E-04	*NCSTN,PSEN1,APH1A,PSENEN*

Supplementary Table 4

Constraint metrics for loss-of-function (LoF) variants from the Genome Aggregation Database (gnomAD; gnomad.broadinstitute.org). **Abbreviations:** expected number of LoF variants (e), observed number of LoF variants (o), probability of intertolerance to LoF variants (pLI), confidence interval (CI). Genes with pLI > 0.9 are considered intolerant to LoF variants.

Gene	e	o	pLI	o/e	90% CI	Intolerant
PSENEN	6.8	0	0.88	0.00	0.00 - 0.44	Yes
APH1A	14.0	2	0.79	0.14	0.06 - 0.45	Borderline
PSEN1	24.4	3	0.97	0.12	0.06 - 0.32	Yes
HEXIM1	12.6	0	0.98	0.00	0.00 - 0.24	Yes
NCSTN	39.1	4	1.00	0.10	0.05 - 0.23	Yes
UBE2M	9.6	1	0.80	0.10	0.04 - 0.49	Borderline
TP53TG3B	n/a	n/a	n/a	n/a	n/a - n/a	n/a
PTPN11	35.2	1	1.00	0.03	0.01 - 0.14	Yes
DAP	5.3	4	0.00	0.76	0.37 - 1.63	No
POLR1A	94.8	16	1.00	0.17	0.11 - 0.26	Yes
CNIH1	7.7	1	0.65	0.13	0.04 - 0.62	Borderline
ZNF792	18.3	16	0.00	0.87	0.59 - 1.33	No
TCEB2	4.7	2	0.09	0.43	0.17 - 1.33	No
TAZ	13.0	2	0.73	0.15	0.06 - 0.48	Borderline
COX15	22.8	21	0.00	0.92	0.65 - 1.33	No
SDAD1	45.5	36	0.00	0.79	0.60 - 1.04	No
LTV1	28.0	16	0.00	0.57	0.39 - 0.87	No
OST4	0.9	0	0.39	0.00	0.00 - 1.74	No
DNA2	51.3	36	0.00	0.70	0.54 - 0.93	No
RPN2	31.4	8	0.11	0.26	0.15 - 0.46	No
TMEM258	4.2	4	0.00	0.95	0.46 - 1.81	No
ALG5	23.2	5	0.46	0.22	0.11 - 0.45	No
DDOST	21.5	9	0.00	0.42	0.25 - 0.73	No
ATP5B	n/a	n/a	n/a	n/a	n/a - n/a	n/a
STT3A	42.3	13	0.00	0.31	0.20 - 0.49	No
TNFRSF17	6.3	8	0.00	1.27	0.72 - 1.88	No

Supplementary Figure 1

Expression of genes identified in the screen across 24 blood cell populations. Data are bulked, imputed single-cell mRNA-sequencing data for 35,582 blood and bone marrow cells from Granja *et al* (Nat Biotechnol. 2019; 37(12): 1458–1465), log2-transformed and median-centered per gene. The genes *RCEB2*, *RPN2*, and *ATP5B* were not represented in this data set.

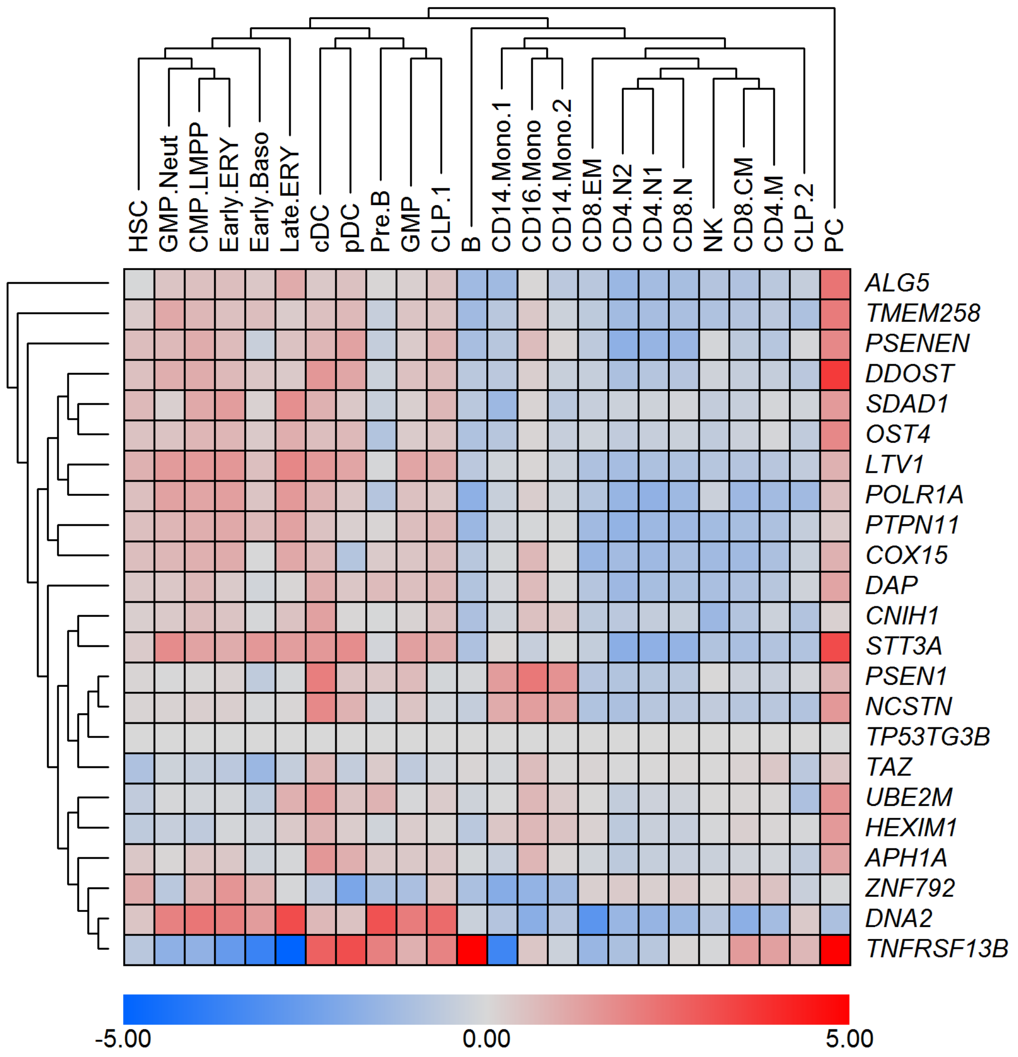

Supplementary Figure 2

Expression of genes identified in the screen across 17 sorted blood cell populations. Data are bulk mRNA-sequencing data from Ulirsch *et al* (Nat Genet. 2019 Apr;51(4):683-693), supplemented with data for plasma cells from Ajore *et al* (Nat Comms. 2022 Jan 10;13(1):151).

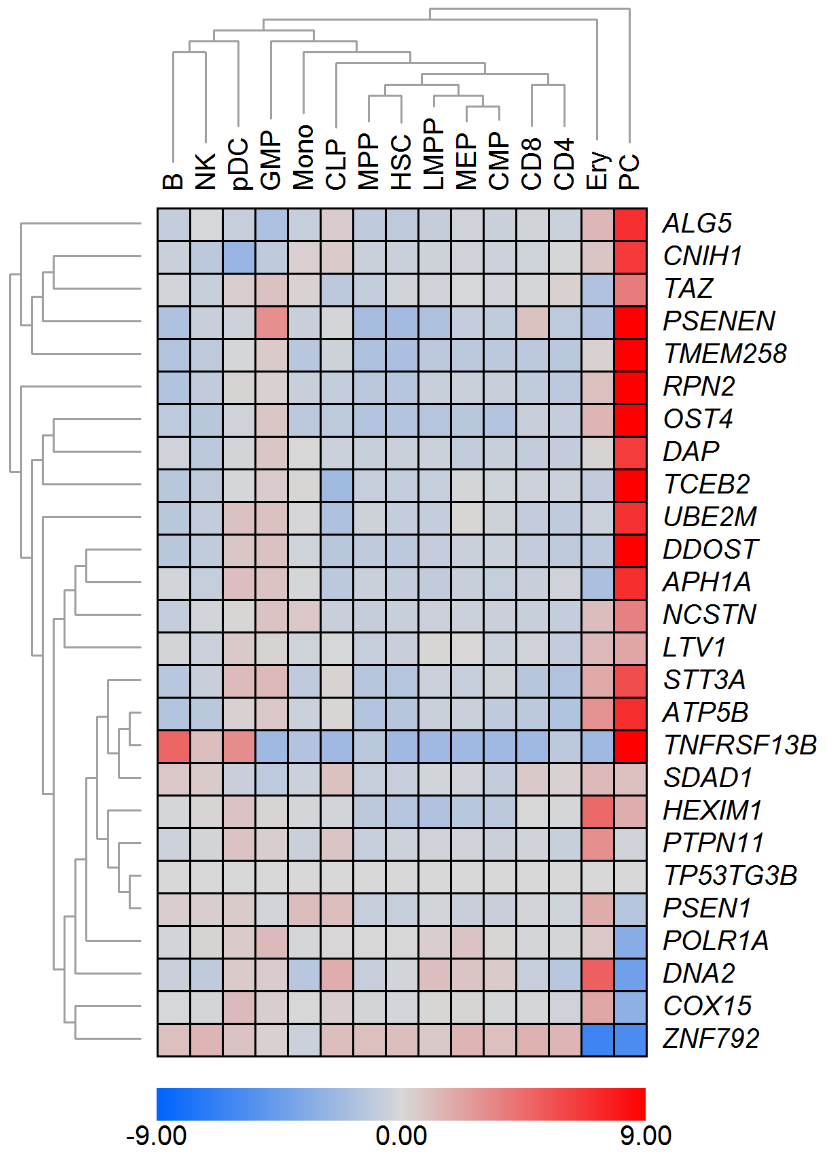

Supplementary Figure 3

Enrichment of expression of candidate genes in different blood cell types in the two gene expression data sets. **(a)** P-values for Student's t-test of normalized expression values for candidate gene for one cell type versus other cell types; **(b)** corresponding results with Wilcoxon rank-sum test.

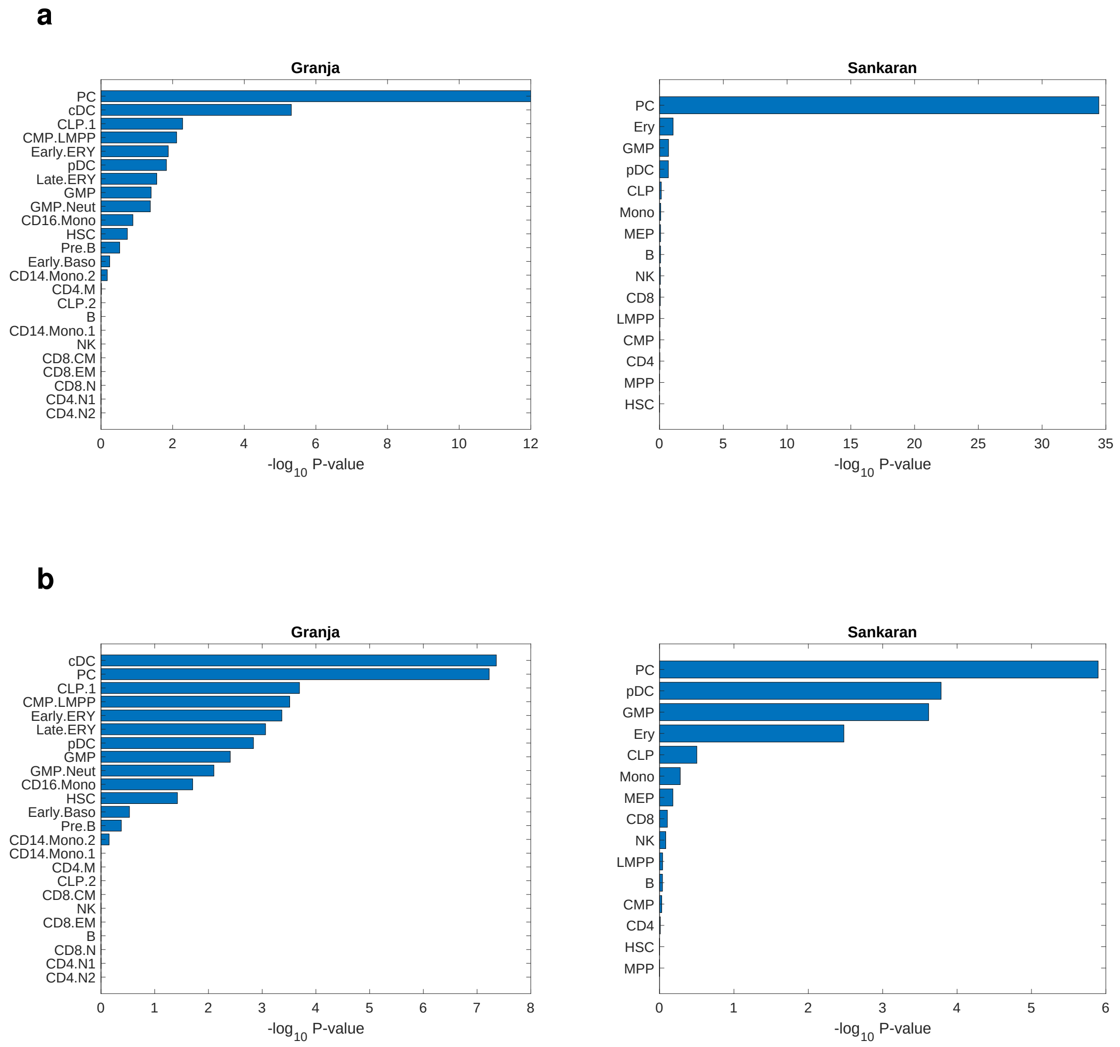

Supplementary Figure 4

Functional interactions between the genes showing strong effects on BCMA expression in our genome-wide CRIPSR/Cas9 screen. Data from STRING database. Edges represent protein-protein associations: curated databases (cyan), experimentally determined (purple), gene neighborhood (green), gene fusions (red), gene co-occurrence (blue), text mining (yellow), co-expression (black), and protein homology (light blue). As shown, two distinct subnetworks are formed by γ-secretase genes and genes involved in protein transport across the endoplasmic reticulum and N-glycolysation.

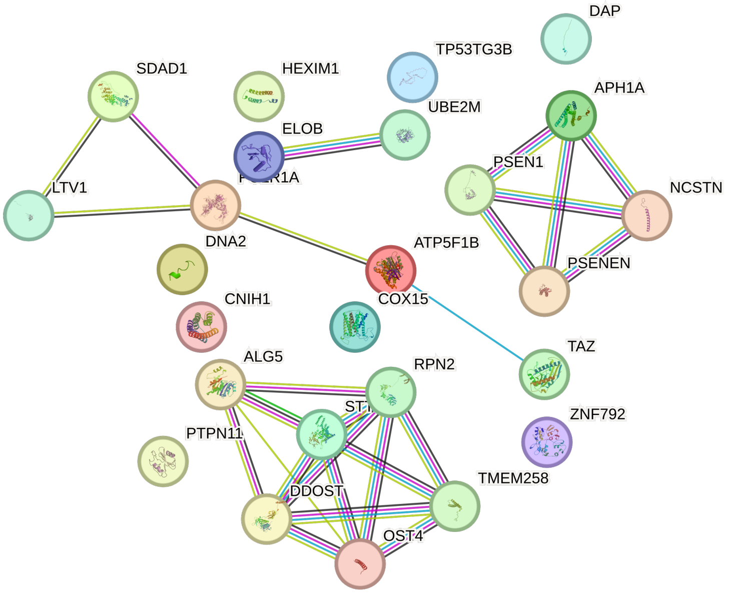

Supplementary Figure 5

For further validation, we performed CRISPR/Cas9 knockdown of selected genes in OPM2 cells and quantified BCMA expression. **(a)** panels: CRISPR/Cas9-treated cells show a bimodal distribution, as only a portion of the cells are edited. Using Gaussian Mixture Modeling, we estimated the mean BCMA intensity of the intensity-shifted cell population (blue dashed) and calculated the log10 fold-changed relative to the mean intensity of the control cells (grey dashed). **(b)**: log10 fold-changes across biological replicates (four per gene); **(c)** and **(d)**: Percentage of positive events in CRISPR-treated vs control cells. Positive events and negative events were defined as cells with BCMA expression higher than, or less than, two standard deviation of the mean BCMA expression of the control cells.

Gamma-secretase genes

PSENEN

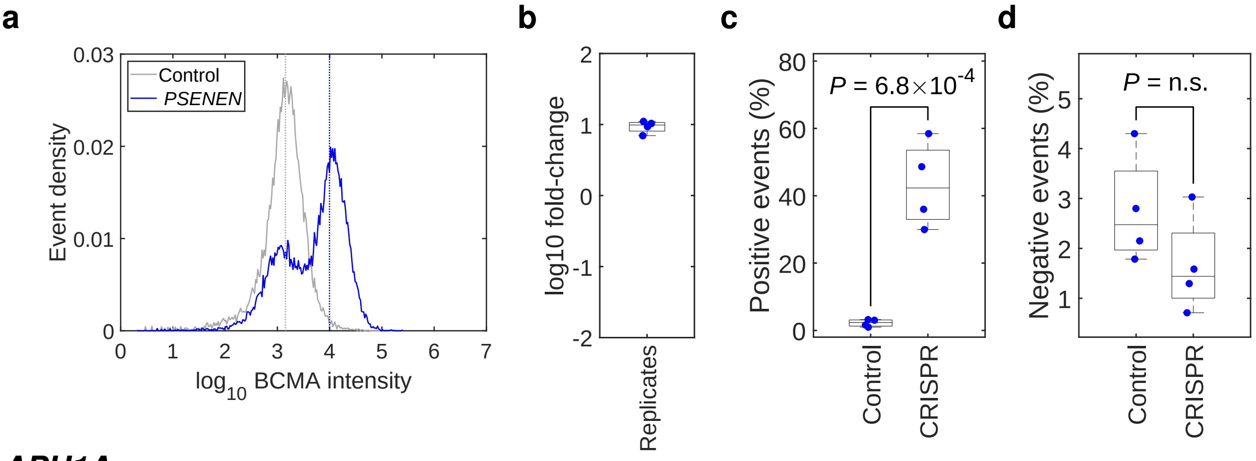

APH1A

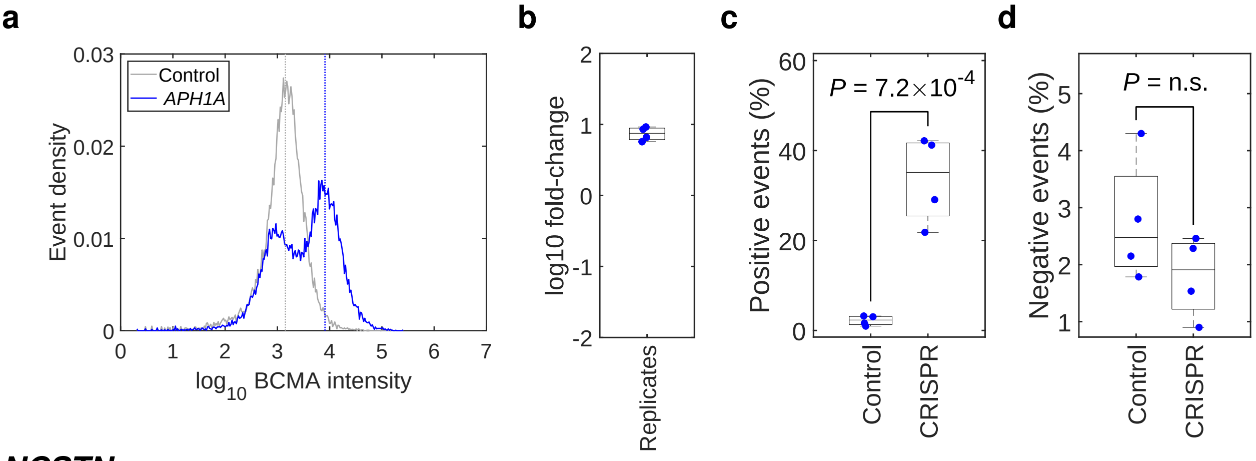

NCSTN

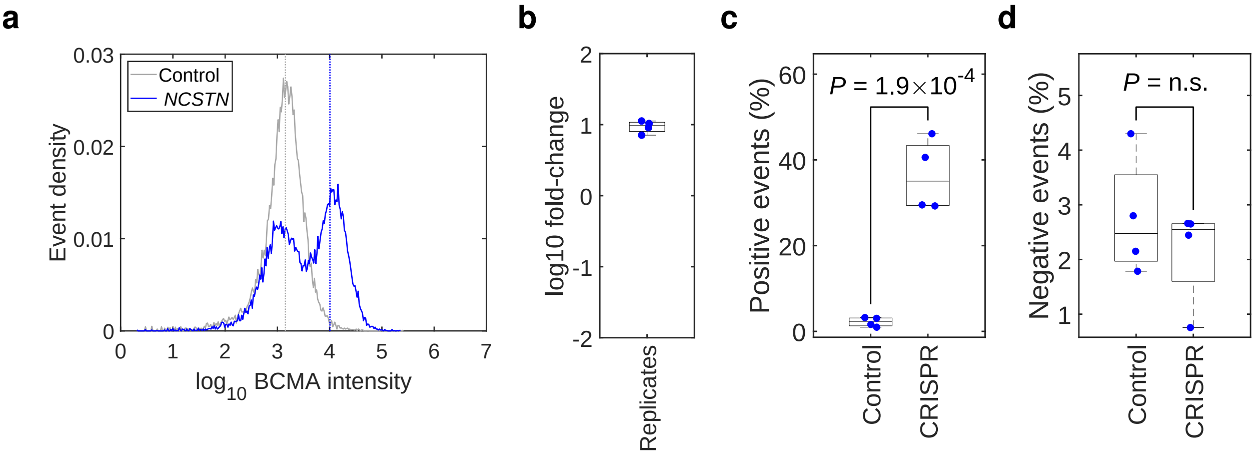

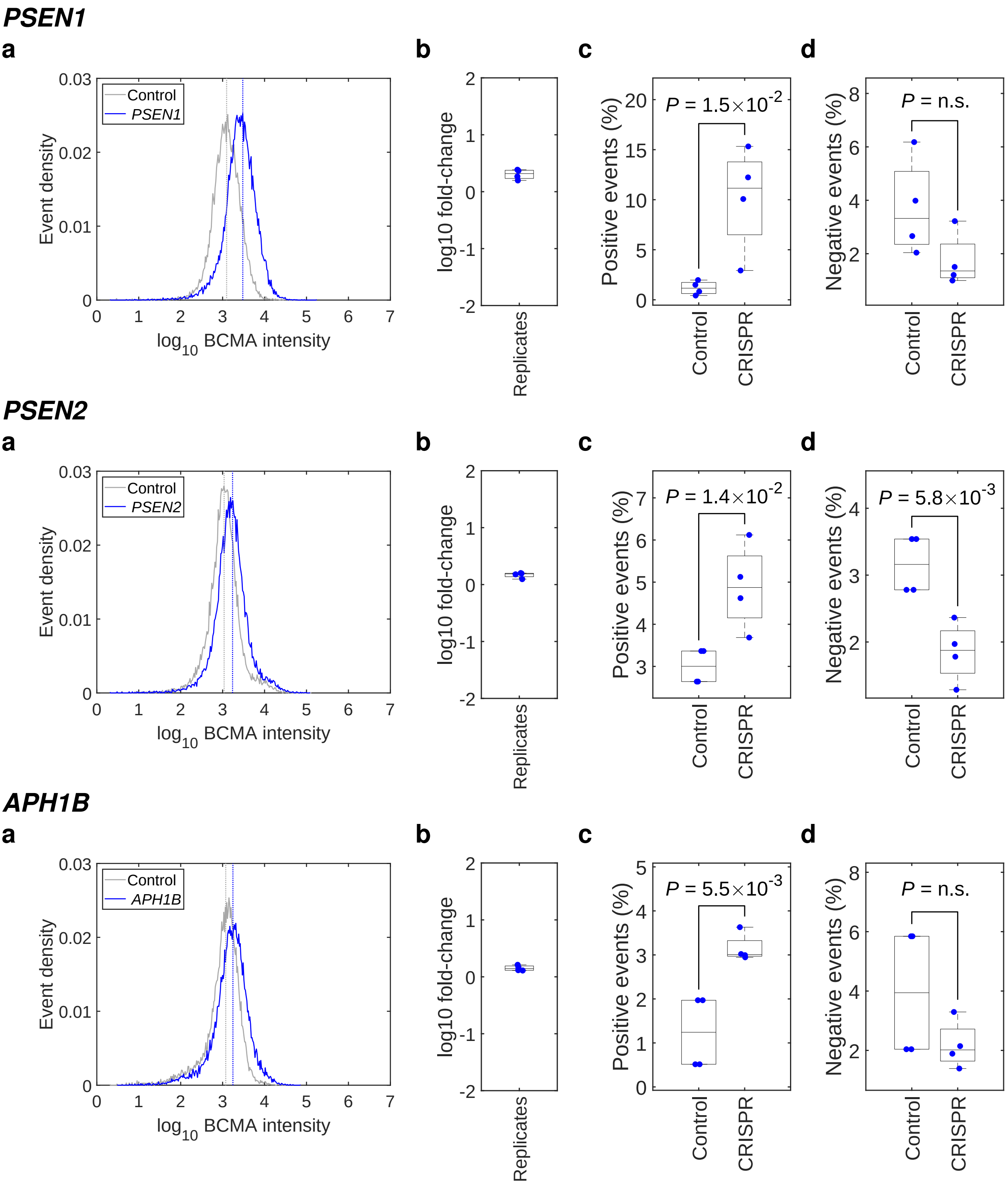

PSEN1
a
Control
PSEN1
Event density
0.03
0.02
0.01
0
0 1 2 3 4 5 6 7
log10 BCMA intensity
b
log10 fold-change
Replicates
c
Positive events (%)
P = 1.5×10-2
Control
CRISPR
d
Negative events (%)
P = n.s.
Control
CRISPR
PSEN2
a
Control
PSEN2
Event density
log10 BCMA intensity
b
log10 fold-change
Replicates
c
Positive events (%)
P = 1.4×10-2
Control
CRISPR
d
Negative events (%)
P = 5.8×10-3
Control
CRISPR
APH1B
a
Control
APH1B
Event density
log10 BCMA intensity
b
log10 fold-change
Replicates
c
Positive events (%)
P = 5.5×10-3
Control
CRISPR
d
Negative events (%)
P = n.s.
Control
CRISPR

Additional negative regulators

HEXIM1

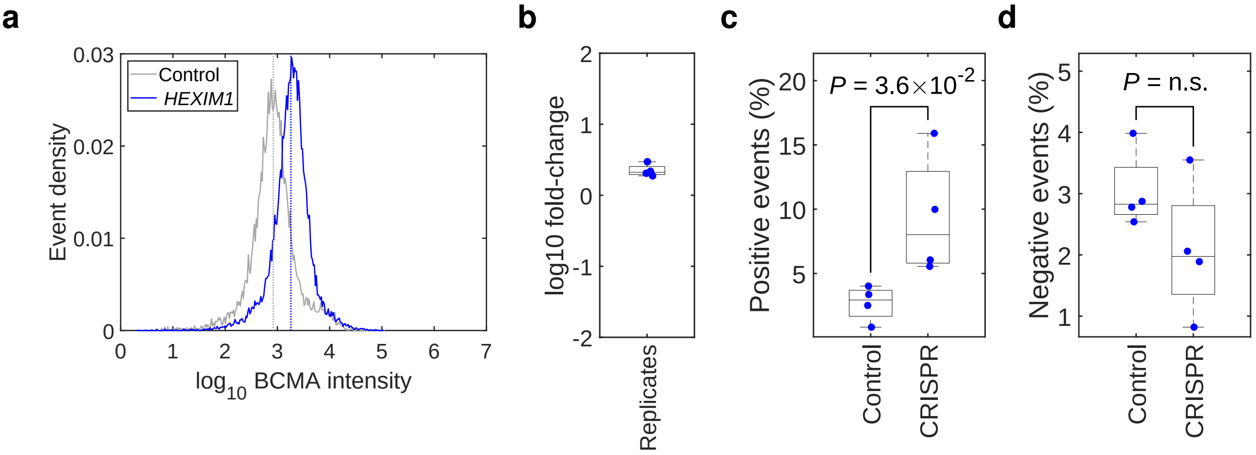

UBE2M

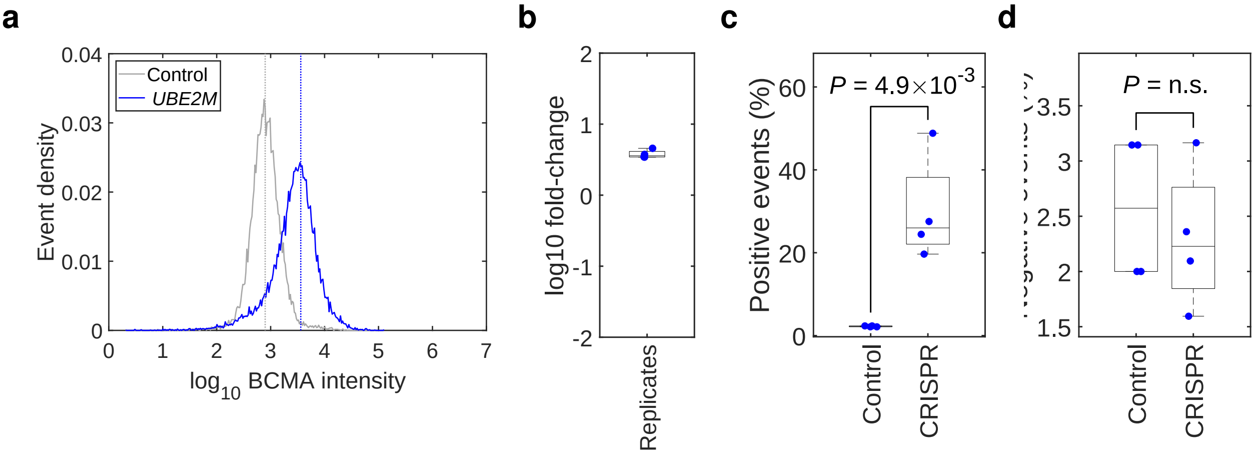

N-glycosylation genes

DDOST

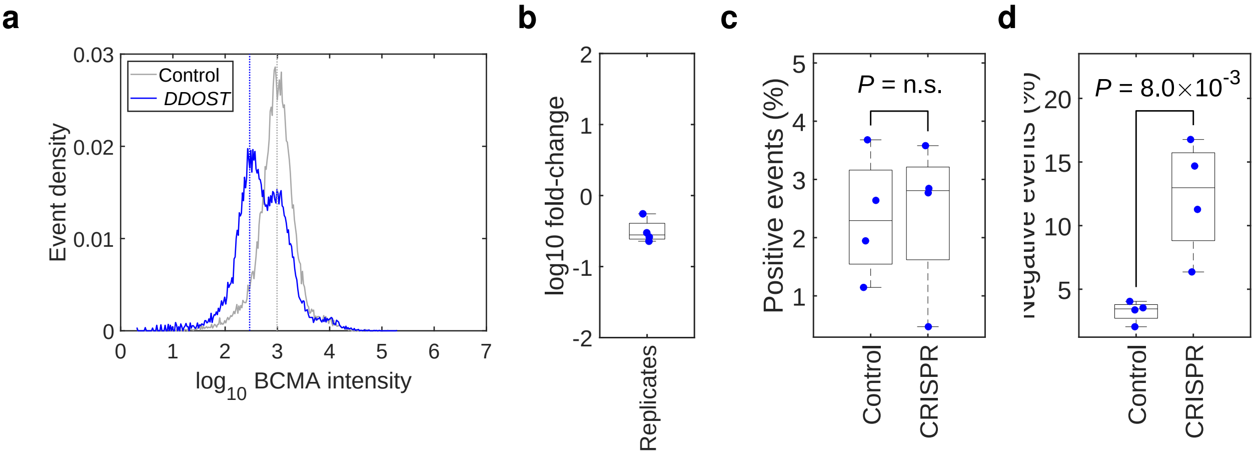

STT3A

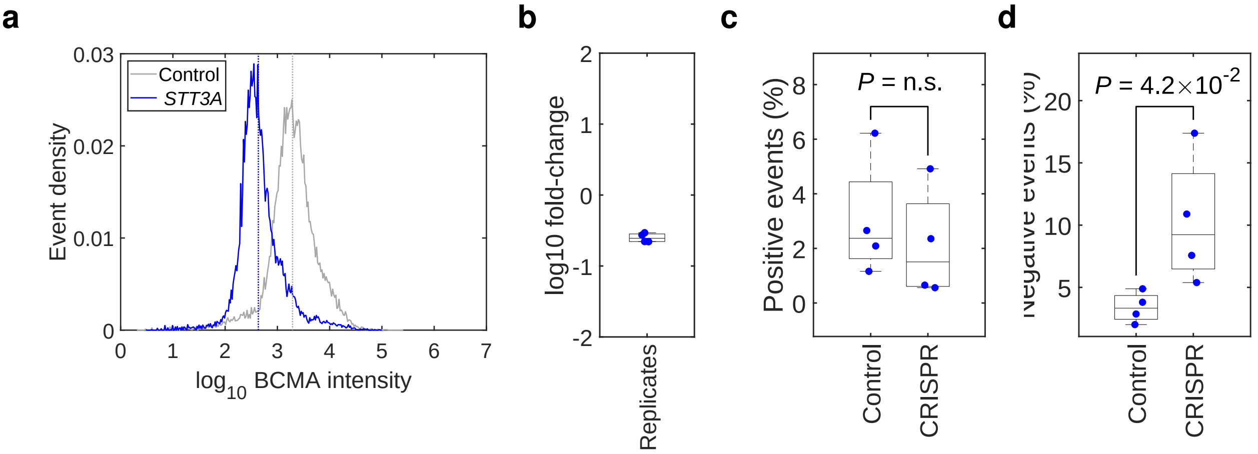

OST4

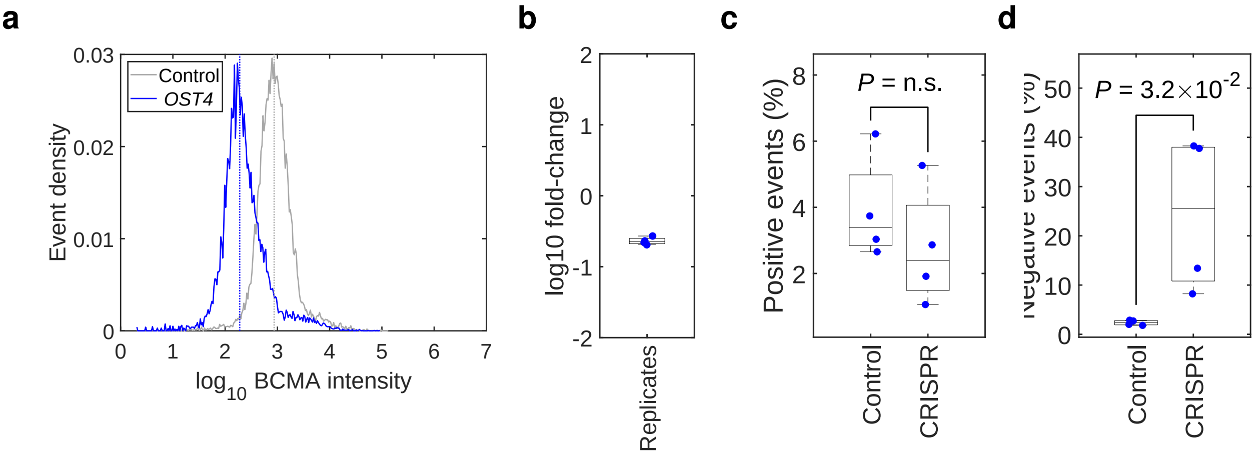

TMEM258

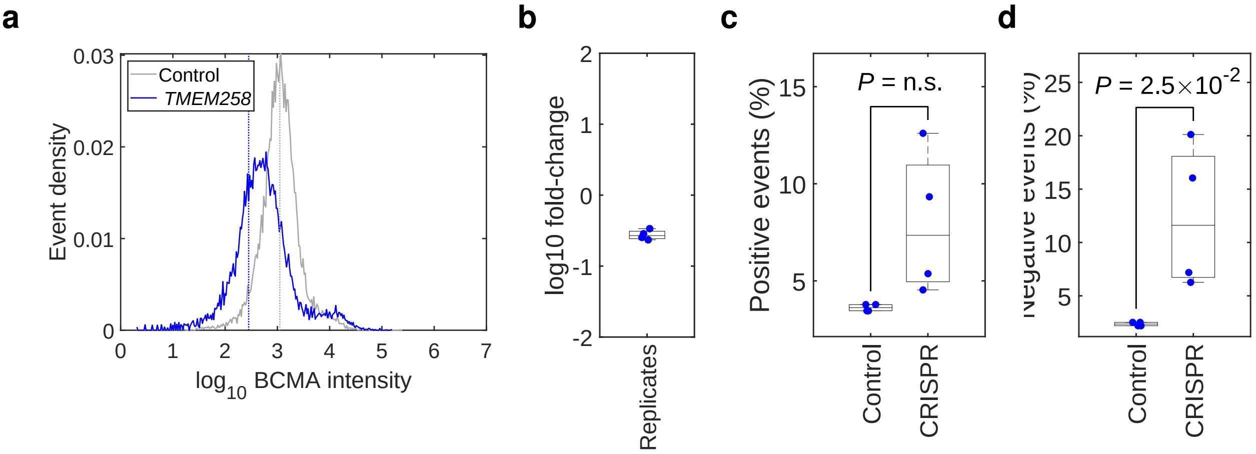

RPN2

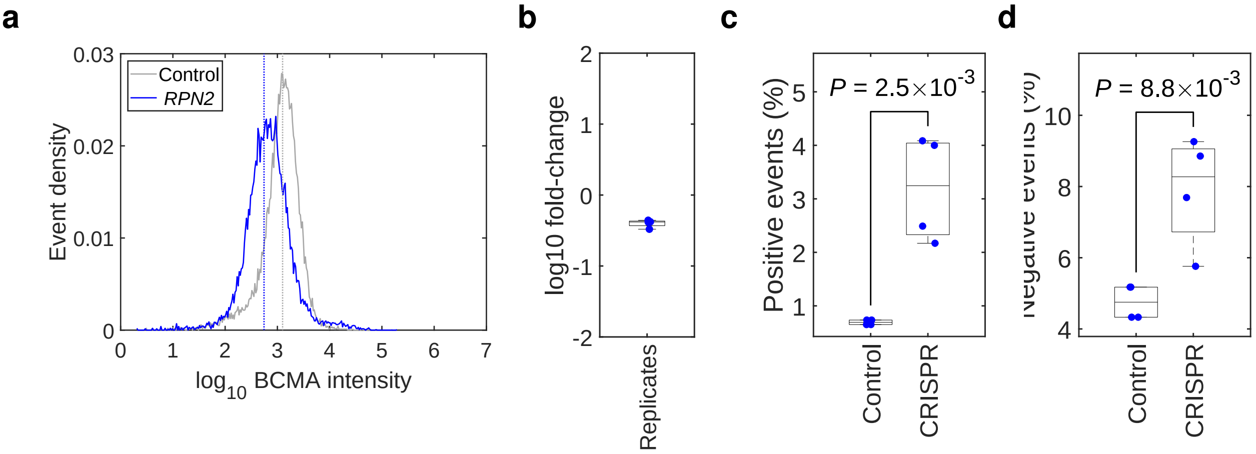

Supplementary Figure 6

Effects of CRISPR/Cas9 knockdown on the growth of 21 MM cell lines in DepMap version 23Q2. Markers indicate Chronos gene effect scores per cell line. Values around zero suggest effects similar to knocking out known non-essential genes. Negative values reaching in the order of -1.0 (red line) indicate essentiality. Genes sorted by median score.

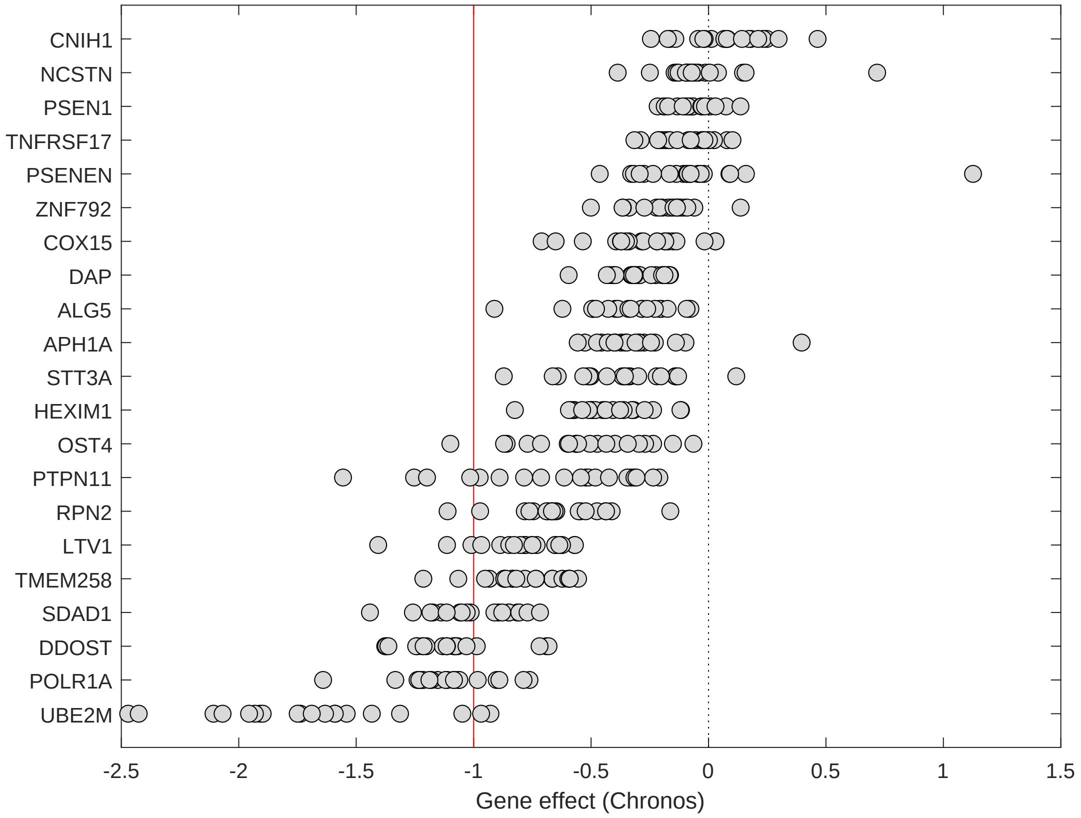

Supplement: Supplementary file 2 — Supplementary Figures and Tables [file 41408_2024_986_MOESM2_ESM.pdf]
